# Simple calculation using anatomical features on pre-treatment verification CT for bladder volume estimation during radiation therapy for rectal cancer

**DOI:** 10.1186/s12885-020-07405-z

**Published:** 2020-10-01

**Authors:** Nalee Kim, Hong In Yoon, Jin Sung Kim, Woong Sub Koom, Jee Suk Chang, Yoonsun Chung

**Affiliations:** 1 Department of Radiation Oncology, Yonsei Cancer Center, Yonsei University Health System, Yonsei University College of Medicine, 50-1 Yonsei-ro, Seodaemun-gu, Seoul, 03722 Republic of Korea; 2 Department of Radiation Oncology, Samsung Medical Center, Sungkyunkwan University School of Medicine, 81 Irwon-ro, Gangnam-gu, Seoul, 06351 Republic of Korea; 3 Department of Nuclear Engineering, Hanyang University, 222 Wangsimni-ro, Seongdong-gu, Seoul, 04763 Republic of Korea

**Keywords:** Bladder volume, Megavoltage CT, Cone-beam CT, Computed tomography simulation

## Abstract

**Background:**

Despite detailed instruction for full bladder, patients are unable to maintain consistent bladder filling during a 5-week pelvic radiation therapy (RT) course. We investigated the best bladder volume estimation procedure for verifying consistent bladder volume.

**Methods:**

We reviewed 462 patients who underwent pelvic RT. Biofeedback using a bladder scanner was conducted before simulation and during treatment. Exact bladder volume was calculated by bladder inner wall contour based on CT images (V_ctsim_). Bladder volume was estimated either by bladder scanner (V_scan_) or anatomical features from the presacral promontory to the bladder base and dome in the sagittal plane of CT (V_ratio_). The feasibility of V_ratio_ was validated using daily megavoltage or kV cone-beam CT before treatment.

**Results:**

Mean V_ctsim_ was 335.6 ± 147.5 cc. Despite a positive correlation between V_ctsim_ and V_scan_ (*R*^2^ = 0.278) and between V_ctsim_ and V_ratio_ (*R*^2^ = 0.424), V_ratio_ yielded more consistent results than V_scan_, with a mean percentage error of 26.3 (SD 19.6, *p* < 0.001). The correlation between V_ratio_ and V_ctsim_ was stronger than that between V_scan_ and V_ctsim_ (Z-score: − 7.782, *p* < 0.001). An accuracy of V_ratio_ was consistent in megavoltage or kV cone-beam CT during treatment. In a representative case, we can dichotomize for clinical scenarios with or without bowel displacement, using a ratio of 0.8 resulting in significant changes in bowel volume exposed to low radiation doses.

**Conclusions:**

Bladder volume estimation using personalized anatomical features based on pre-treatment verification CT images was useful and more accurate than physician-dependent bladder scanners.

**Trial registration:**

Retrospectively registered.

## Background

Radiation therapy (RT) to the whole pelvis often induces several acute or late gastrointestinal toxicities such as abdominal pain, diarrhea, small bowel obstruction, or perforation in locally advanced rectal cancer patients receiving preoperative concurrent chemoradiotherapy (CRT) [1, 2]. Thus, many clinicians have attempted to reduce RT doses and irradiated volumes using non-surgical methods, including the prone position, small bowel displacement devices, and bladder distension [1, 3–6].

Studies show that there is a large variation in bladder volumes throughout treatment. To account for bladder filling, a sufficiently large margin can be applied. Otherwise, physicians provide instructions to maintain consistent bladder distension and to regularly check bladder volume during preoperative CRT. However, daily verification of full bladder status in clinical practice is challenging. A portable automated ultrasonic bladder scanner is usually used for estimating bladder volume. Protocol-based maintenance using ultrasonic bladder scanners is employed at our institution; it is reported that bladder volume can be maintained more consistently during RT. [3, 7] However, in clinical practice, accuracy is the main issue with this method due to deviations introduced by physicians performing bladder scans and the types of bladder scanners used. Thus, the most accurate and practical bladder volume estimation procedure needs to be identified for obtaining consistent results to avoid physicians and patients from receiving inaccurate bladder volume information.

In the era of intensity modulated radiation therapy (IMRT) and image-guided radiation therapy (IGRT), routine mega-voltage CT (MVCT) or kilovoltage cone-beam CT (kVCBCT) is performed immediately before each treatment session. If bladder volumes can be estimated using MVCT or kVCBCT findings, an additional procedure for estimating bladder volume can be avoided. Furthermore, the image quality of MVCT or kVCBCT allows for the visualization of pelvic organs such as the bladder, colon, rectum, uterus, prostate, bones, etc. We hypothesized that estimation of bladder volume using unaffected anatomical features on MVCT or kVCBCT images would be a practical and accurate method; thus, we aimed to investigate our first hypothesis by conducting a retrospective study.

## Methods

### Patient population

Following the Health Institutional Review Boards of Yonsei University Hospital (No. 4–2019-0887) approval, we retrospectively reviewed the data of patients diagnosed with rectal cancer from September 2012 to January 2018. Then, we screened for patients treated with RT under contrast enhanced planning CT with 3 mm thickness (*n* = 499). We excluded patients who received RT with a palliative aim without considering bladder volume, those without bladder scan data, those with rectal ballooning, those who underwent cystectomy, and those who had anatomic distortions after lumbar spine surgery. After applying these exclusion criteria, we ultimately analyzed data of 462 patients. The requirement for informed consent was waived owing to the study’s retrospective nature.

### CT simulation and bladder volume estimation

CT simulation was performed by positioning the patient with a full bladder in either the prone position with a belly board or in the supine position. All the patients were asked to follow the institutional bladder-filling protocol before CT simulation. Bladder volume estimation using bladder scan (V_scan_) was performed using a portable automated ultrasonic bladder scanner (Bicon-700, Mcube Technology, Korea) during simulation. Physicians placed the bladder scanner on the suprapubic area and angled the scanner towards the bladder with the patient lying in the supine position. Physicians consecutively measured bladder volumes five times; the mean value was recorded as V_scan_.

To calculate bladder volumes based on ratio of anatomical features (V_ratio_), the CT simulation isocenter was used as the reference point. The typical isocenter was located at the midline with a mid-depth of 2 cm above the upper margin of the femur head. With sagittal view through the isocenter, we measured the distance from the sacral promontory to the bladder dome (U) and from the sacral promontory to the bladder base (L). Then we calculated the ratio for height of bladder as follows:

$$ \mathrm{Ratio}\ \mathrm{of}\ \mathrm{bladder}\ \mathrm{height}=\frac{\left(L-U\right)}{L} $$

Then, equation for V_ratio_ was conferred by linear regression using the ratio of bladder height (Additional file 1: Figure S1): V_ratio_ = 39.78 + 312.13 * (Ratio of bladder height).

**Fig. 1:**
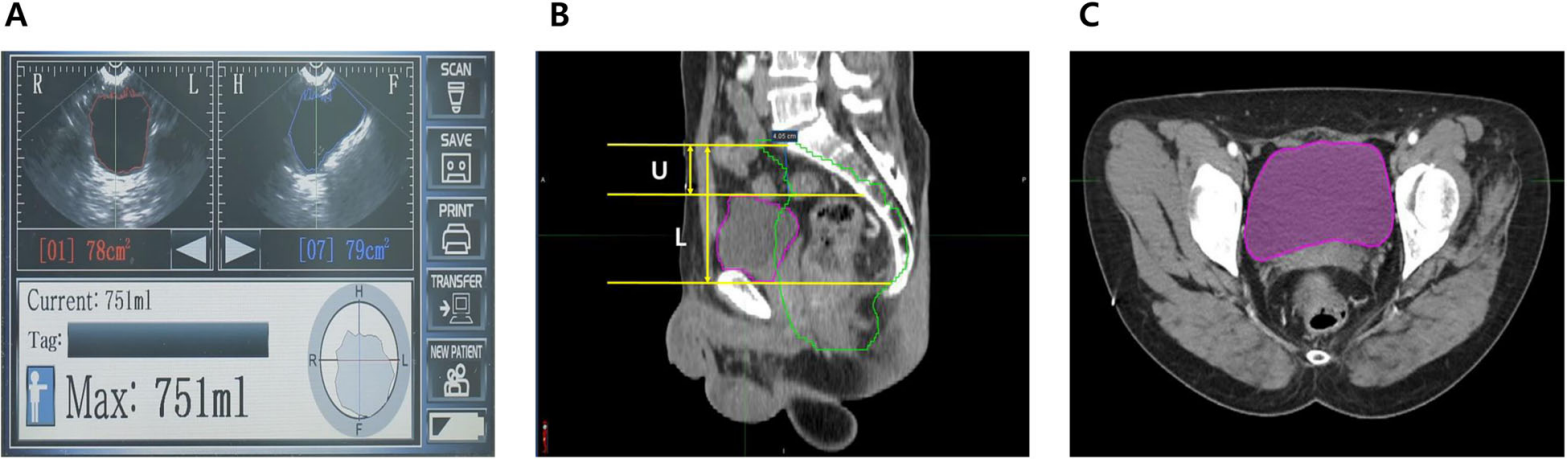
Methods of bladder volume estimation: V_scan_ (**a**), ratio of bladder height (**b**), and V_ctsim_ (**c**)

After CT simulation, the inner bladder wall was contoured from the base to the dome of the bladder for calculating the simulation bladder volume (V_ctsim_) using MIM software (MIM Software Inc., Cleveland, OH, USA). While contouring the inner bladder wall, CT images were viewed using a mediastinal window (window width/level [Hounsfield Unit] = 500/39) setting. Since actual bladder volume measurement is practically impossible, V_ctsim_ was considered as the actual bladder volume. Each method for bladder volume estimation (V_scan_, V_ratio_, and V_ctsim_) is illustrated in Fig. 1.

We performed further validation using MVCT and kVCBCT images (total 108 sets) of 18 patients treated from December 2017 to January 2018. Similar to the volume estimation method used in CT simulation, the inner bladder wall was contoured from the base to the dome of the bladder to calculate bladder volume in MVCT/kVCBCT using MIM software. After our preliminary analysis, patients were sent back to the waiting room after insufficient image verification for bladder volume estimation using MVCT/kVCBCT instead of waiting for repeated error prone bladder scans. After 10–15 min of drinking 100 cc of water, repeated MVCT/kVCBCT verification was performed before treatment. Most patients underwent just one additional MVCT/kVCBCT after insubstantial bladder volume verification during the first time. With sufficient bladder volume verification using MVCT/kVCBCT, patients underwent each treatment with positive feedback for maintaining consistent bladder volume.

### Statistical analysis

To simplify the comparison, we considered 1 cm^3^ on the simulation CT to be equivalent to 1 mL on the bladder scan. To assess the accuracy of each estimation tool, the correlations between V_scan_ and V_ctsim_ and between V_ratio_ and V_ctsim_ were analyzed using Pearson’s correlation coefficient. The Steiger’s Z-test was used for comparing the correlation coefficients. Each coefficient derived from the correlation between V_ratio_ and V_ctsim_ and between V_scan_ and V_ctsim_ was used to generate the final bladder volume estimation equation. After deriving an equation for estimating bladder volume, we determined percentage differences between V_scan_ and V_ratio_ using standard deviations (SDs) and 95% confidence intervals (CIs). We also performed the Z-test after Fisher’s transformation to evaluate differences among subgroups (sex, position). A *p*-value < 0.05 was considered to be statistically significant. All analyses were performed using GraphPad Prism (version 8.00 for Windows; GraphPad Software, La Jolla California, USA) and R (version 3.2.2; R Foundation for Statistical Computing, Vienna, Austria).

## Results

We analyzed 462 consecutive CRT-receiving rectal cancer patients who underwent bladder scans. Most patients (88.7%) underwent CT simulation in the prone position. Mean actual bladder volume from V_ctsim_ was 335.6 mL. Other characteristics of the whole study population are listed in Table 1.

**Table 1:** Patient and baseline characteristics

	N	%
Sex		
Female	194	42.0
Male	268	58.0
Position		
Prone	410	88.7
Supine	52	11.3
	Mean ± SD	
Age (yr)	59.1 ± 12.7	
V_scan_ (mL)	236.0 ± 112.4	
V_ctsim_ (mL)	335.6 ± 147.5	
	Median (range)	
Anatomic features (cm)		
Upper limit (U)	0.99 (−7.69–9.25)	
Lower limit (L)	10.03 (5.61–14.18)	
Ratio (L-U/L)	0.91 (0.19–2.08)	

*Abbreviations: yr* year, *SD* standard deviation, *V*_*ctsim*_ bladder volume measurement using computed tomography simulation images

**Fig. 2:**
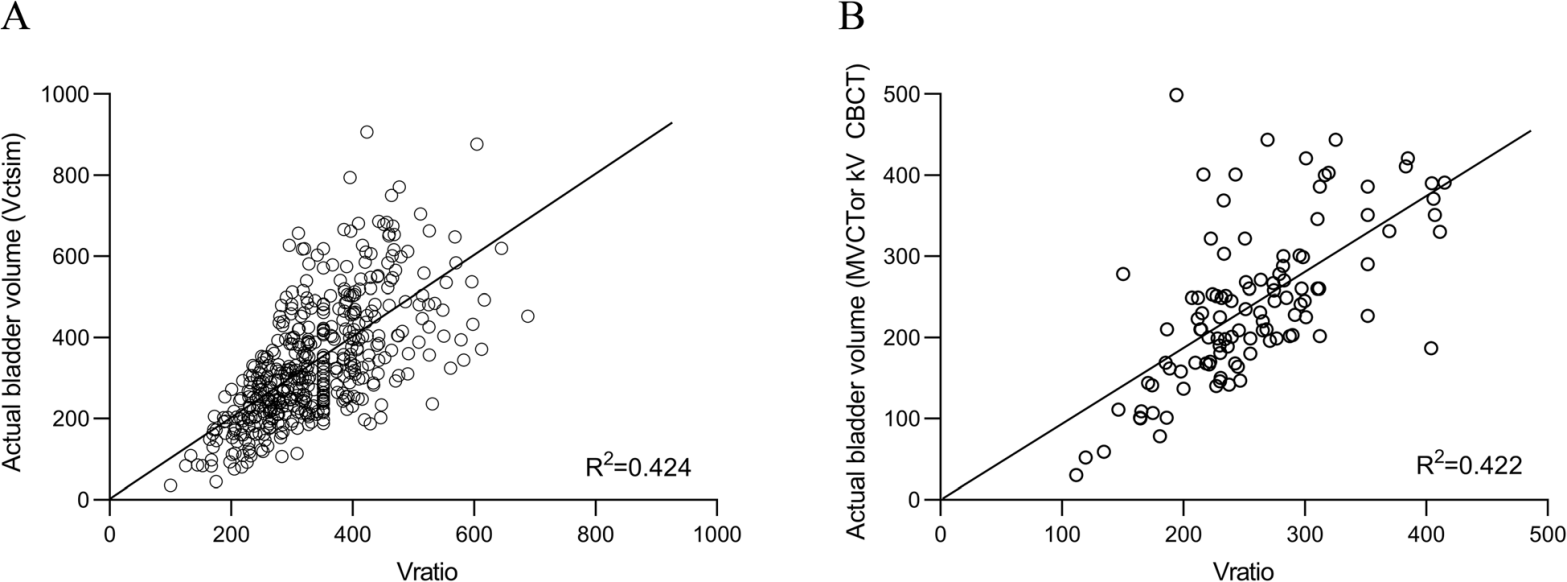
Scatter plots showing the correlation between simulation bladder volume (V_ctsim_) and bladder volume from the ratio of bladder height based on anatomical features (V_ratio_) from CT simulation images (**a**) and between bladder volume delineated in megavoltage CT (MVCT) and kV cone-beam CT (kV CBCT) and bladder volume estimated by ratio of bladder height (V_ratio_)

Estimated volumes using both the tools (V_ratio_ or V_ctsim_) showed positive correlations with V_ctsim_. Although there was a correlation between V_ratio_ and V_ctsim_ (*R*^2^ = 0.424, Fig. 2a) and V_scan_ and V_ctsim_ (*R*^2^ = 0.278, Additional file 2: Figure S2), we found that V_ctsim_ can be predicted better by using V_ratio_ than by using V_scan_ (Z-score: 2.711, *p* = 0.007) after Steiger’s Z-test. Additionally, V_ratio_ yielded more consistent results than did V_scan_, with a mean percentage error of 26.3 (SD, 19.6; 95% CI, 24.6–28.1; *p* < 0.001; Table 2) to V_ctsim_.

**Table 2:** Comparison between bladder volumes estimated using bladder scan and using anatomic references on planning computed tomography

Estimation tool	Number of data sets	Mean percent difference	95% CI	SD of percent difference	*P*
V_scan_	462	42.7	39.4–45.9	35.5	< 0.001
V_ratio_	462	26.3	24.6–28.1	19.6	

*Abbreviations: CI* confidence interval, *SD* standard deviation, *V*_*scan*_ bladder volume measurement using bladder scan, *V*_*ratio*_ bladder volume measurement using ratio of anatomical features

We found that V_ratio_ differed according to treatment position and sex. V_ratio_ also showed a positive correlation with V_ctsim_ in each subgroup. Additionally, we identified no difference in the degree of correlation between V_ratio_ and V_ctsim_ after performing Steiger’s Z-test with Fisher’s transformation (Additional file 3: Table S1).

**Fig. 3:**
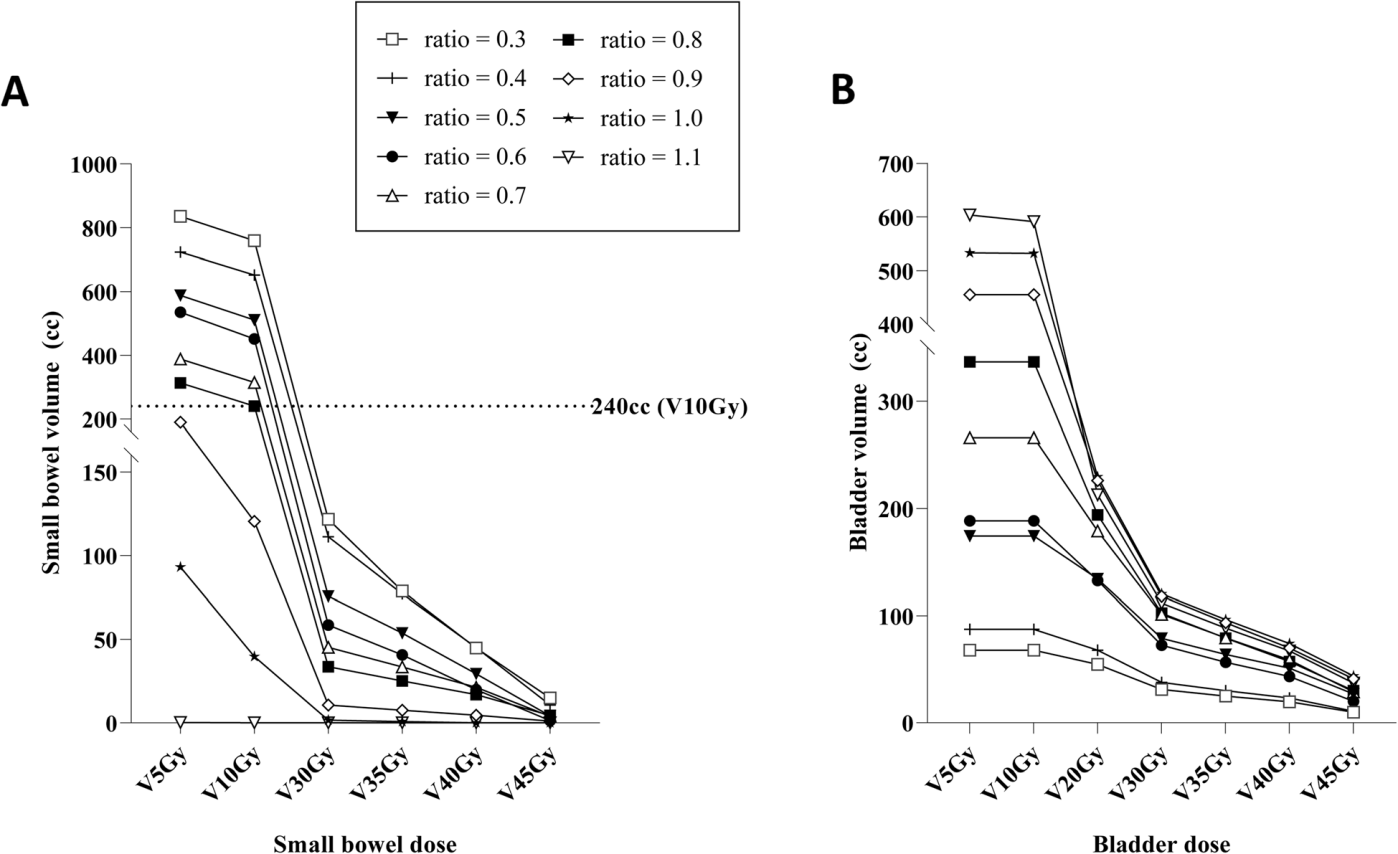
Dose distribution of small bowel (**a**) and bladder (**b**) according to the anatomical ratio in a representative case

We further validated the feasibility of V_ratio_ using MVCT/kVCBCT images. There was no difference in bladder volume estimation based on either the equation derived from CT simulation or the volume estimation equation generated from 108 MVCT/kVCBCT image sets (Z-score: 0.696, *p* = 0.486). Therefore, we adopted the equation from the initial V_ratio_ (from CT simulation) to further verify the feasibility of V_ratio_ in MVCT/kVCBCT images (Fig. 2b). On performing Steiger’s Z-test, we found that V_ratio_ showed a higher degree of consistency in MVCT/kVCBCT bladder volume than did V_scan_ (Z-score: 2.013, *p* = 0.044). Additionally, the mean percent differences between V_ratio_ and actual bladder volume on MVCT/kVCBCT and between V_ratio_ and V_scan_ were 23.4 and 33.2% (*p* < 0.001, Table 3), respectively. Additionally, a representative case revealed that the ratio of 0.8 (V_ratio_ = 289.5) resulted in small bowel V_10Gy_ of 240 cc with a wide variation in the low dose area of the small bowel and bladder in accordance with the relative ratio of anatomical references (Fig. 3a-b).

**Table 3:** Comparison between bladder volumes estimated using bladder scan and using anatomical features on cone-beam computed tomography

Estimation tool	Number of data sets	Mean percent difference	95% CI	SD of percent difference	P
V_scan_	108	33.2	28.5–37.8	26.7	< 0.001
V_ratio_	108	23.4	21.3–29.5	21.4	

*Abbreviations: CI* confidence interval, *SD* standard deviation, *V*_*scan*_ bladder volume measurement using bladder scan, *V*_*ratio*_ bladder volume measurement using ratio of anatomical features

## Discussion

Our findings demonstrated that anatomical features identified on CT (V_ratio_) were more useful for predicting bladder volume than were findings of bladder scans (V_scan_). Our results also suggest that determining the V_ratio_ using MVCT or kV CBCT findings could be more useful and practical than using bladder scans during treatment in routine practice.

Bladder filling is critical for reducing radiation-induced pelvic toxicities, such as enteritis and cystitis, in patients receiving whole-pelvic RT. [8, 9] Modern techniques, such as IMRT and IGRT, can deliver smaller irradiation doses to the small bowel, resulting in less acute gastrointestinal toxicities in patients receiving pelvic RT. [10, 11] The NRG/RTOG 1203 study showed that pelvic IMRT for cervical and endometrial cancer was related with significantly less gastrointestinal and urinary toxicities than was standard RT, based on patient-reported toxicities according to the Expanded Prostate Cancer Index Composite [10]. Despite this, 33.7% of patients receiving IMRT still developed frequent or almost constant diarrhea in the NRG/RTOG 1203 study. Some studies have demonstrated the protective effects of bladder filling on the small bowel in patients receiving IMRT [12, 13]. Recently, Chen et al. showed that the small bowel volume that received 45 Gy was larger when IMRT was delivered on an empty bladder than when IMRT was delivered on a full bladder [12]. Furthermore, recent meta-analysis reported that grade 3 or more small bowel toxicity was related to multiple parameters in small dose area (range, 5–35 Gy) rather than traditional parameters related to 45 Gy [14]. Adequate bladder filling can reduce the small bowel volume affected by small dose area. Therefore, the importance of bladder filling has not diminished, even in the modern RT era of IMRT.

Nevertheless, intra- or inter-fractional variation in bladder volume can cause critical treatment accuracy-related issues. Some studies have reported that the variation in bladder volume can influence changes in target volume and irradiated dose during pelvic RT. [8, 15, 16] Consistent results of volume measurement are indispensable to ensure effective and safe advanced-RT techniques. Thus, many studies have investigated accurate and useful tools for the regular measurement of bladder volumes. Among several methods, bladder ultrasonography is known to be an easy and useful method [3, 16]. As previously described, a biofeedback protocol based on the correlation between ultrasonography scan and bladder volume resulted in consistent bladder volume maintenance and better performance than did a self-controlled maintenance program [3, 7]. However, with the accumulation of clinical experience, several problems have been identified in bladder volume estimation in clinical practice. Following are the reasons for accuracy issues related to bladder scans: first, bladder scan results are highly dependent on the performing physicians and second, while bladder scan results might be representative of the actual bladder volume, these results are not equal to the actual bladder volumes. Thus, we attempted to identify more accurate and useful measurement tools that are unaffected by the performing physicians and decided to evaluate the feasibility of bone anatomical features for estimating bladder volumes. For instance, there is a wide variation in small bowel dose distribution according to the ratio of bladder height. (Fig. 3a). Additionally, in a representative case, we observed that an anatomical ratio > 0.8 could be a surrogate for minimizing the small bowel irradiated volume. Further investigation on the optimal cut-off of anatomical variance could help technicians and physicians to treat patients more effectively.

Based on our study results, bony anatomical reference points on CT images could be more accurate and useful for estimating bladder volumes, as they are not influenced by external factors. We investigated the availability of anatomical reference points on MVCT/kVCBCT images because MVCT/kVCBCT is considered easily applicable in clinical practice; furthermore, if MVCT/kVCBCT can be utilized, additional procedures for measuring bladder volumes before treatment are not necessary because MVCT/kVCBCT can be performed daily for each patient. Given our findings, our hypothesis appears to be well-validated, and the use of anatomical features on MVCT/kVCBCT images could not only be accurate and useful but also less labor-intensive and time-consuming; this is because bladder volumes can be immediately determined by measuring the distances between anatomical features on the images obtained for patient setup before each treatment.

It has been questioned whether the image quality of MVCT/kVCBCT with respect to contrast and resolution allows the identification of anatomical features and differentiation of the bladder from other pelvic organs. However, MVCT/kVCBCT is sufficiently useful for identifying bony anatomical structures and for distinguishing the bladder from other pelvic organs. MVCT has already been established for the purposes of patient setup and dose verification in IGRT delivered using helical tomotherapy (Accuray, Sunnyvale, CA) [17, 18]. kVCBCT is also known to be useful for improving the accuracy of the set-up and for continuously monitoring changes in tumor volume and normal organs, making it possible for kVCBCT to be used in adaptive RT [19–21].

The retrospective study design is a limitation of this study. However, the purpose of our study was to validate our hypothesis for overcoming the limitations of bladder scans for bladder volume estimation, which is an established routine practice, based on our clinical experiences. We think that our results are sufficient for demonstrating the feasibility of bladder volume estimation using anatomical features based on validation using MVCT/kVCBCT findings. In addition, bladder volumes affected by low dose (range, 5–10 Gy) could increase with absolute volume but not a proportional volume of bladder (Additional file 4: Figure S3) while preventing small bowel damage. Such yin and yang should be cautiously considered by physicians.

## Conclusions

Our findings demonstrated that bladder volume estimation using personalized anatomical features on CT simulation or cone-beam CT was clinically useful in terms of assessment accuracy and simple implementation. Anatomical ratio could be a promising surrogate for adequate bladder filling in further clinical implementation. Bladder volume estimation at that time of image verification using MVCT/kVCBCT could help physicians and patients during pelvic RT.

## Supplementary information

**Additional file 1: Figure S1.** Scatter plots showing the correlation between simulation bladder volume (V_ctsim_) and the ratio of bladder height based on anatomic reference.

**Additional file 2: Figure S2.** Scatter plots showing the correlation between simulation bladder volume (V_ctsim_) and bladder volume based on bladder ultrasonography scan (V_scan_).

**Additional file 3: Table S1.** Correlation between bladder volume and baseline characteristics.

**Additional file 4: Figure S3.** Dose-volume histogram of small bowel and bladder according to the anatomical ratio in a representative case.

## Data Availability

The datasets generated and analyzed during the current study are not publicly available due to institutional data protection law and confidentiality of patient data but are available from the corresponding author on reasonable request in person.

## Abbreviations

RT: radiation therapy
CRT: chemoradiotherapy
IMRT: intensity modulated radiation therapy
IGRT: image-guided radiation therapy
MVCT: mega-voltage CT
kVCBCT: kilovoltage cone-beam CT
SD: standard deviation
CI: confidence interval
