# Supplementary material for: Simple calculation using anatomical features on pre-treatment verification CT for bladder volume estimation during radiation therapy for rectal cancer
Source: BMC Cancer. 2020 Oct 1;20:942. doi: 10.1186/s12885-020-07405-z (PMC7528380; doi:10.1186/s12885-020-07405-z)

**Additional file 1: Figure S1.** Scatter plots showing the correlation between simulation bladder volume (V_ctsim_) and the ratio of bladder height based on anatomic reference.


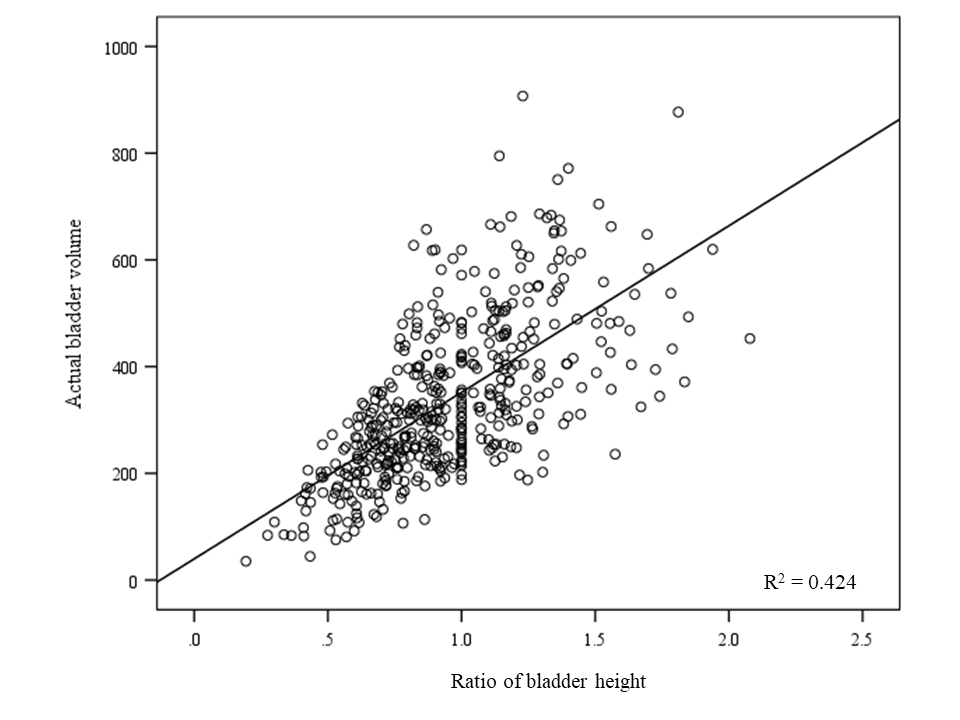

Supplement: Supplementary file 1 — Additional file 1: Figure S1. Scatter plots showing the correlation between simulation bladder volume (Vctsim) and the ratio of bladder height based on anatomic reference. [file 12885_2020_7405_MOESM1_ESM.docx]
