# Supplementary material for: Simple calculation using anatomical features on pre-treatment verification CT for bladder volume estimation during radiation therapy for rectal cancer
Source: BMC Cancer. 2020 Oct 1;20:942. doi: 10.1186/s12885-020-07405-z (PMC7528380; doi:10.1186/s12885-020-07405-z)

**Additional file 2: Figure S2**. Scatter plots showing the correlation between simulation bladder volume (V_ctsim_) and bladder volume based on bladder ultrasonography scan (V_scan_).


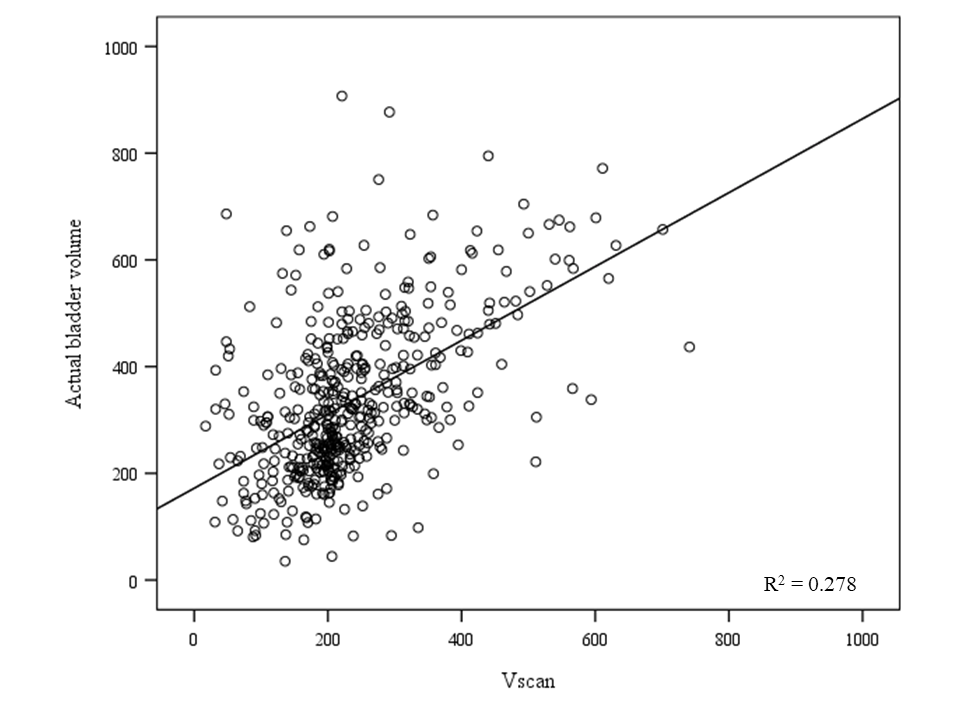

Supplement: Supplementary file 2 — Additional file 2: Figure S2. Scatter plots showing the correlation between simulation bladder volume (Vctsim) and bladder volume based on bladder ultrasonography scan (Vscan). [file 12885_2020_7405_MOESM2_ESM.docx]
