# Supplementary material for: Simple calculation using anatomical features on pre-treatment verification CT for bladder volume estimation during radiation therapy for rectal cancer
Source: BMC Cancer. 2020 Oct 1;20:942. doi: 10.1186/s12885-020-07405-z (PMC7528380; doi:10.1186/s12885-020-07405-z)
Supplement: Supplementary file 3 — Additional file 3: Table S1. Correlation between bladder volume and baseline characteristics. [file 12885_2020_7405_MOESM3_ESM.docx]

**Additional file 3: Table S1.** Correlation between bladder volume and baseline characteristics

|  | N | Pearson’s correlation coefficient (r) | *P* | Z test after Fisher transformation | *P* |
| --- | --- | --- | --- | --- | --- |
| Female | 194 | 0.732 | <0.001 | 1.570 | 0.058 |
| Male | 268 | 0.655 | <0.001 |  |  |
| Supine | 52 | 0.668 | <0.001 | 0.628 | 0.265 |
| Prone | 410 | 0.612 | <0.001 |  |  |
