# Supplementary material for: Simple calculation using anatomical features on pre-treatment verification CT for bladder volume estimation during radiation therapy for rectal cancer
Source: BMC Cancer. 2020 Oct 1;20:942. doi: 10.1186/s12885-020-07405-z (PMC7528380; doi:10.1186/s12885-020-07405-z)

**Additional file 4: Figure S3**. Dose-volume histogram of small bowel and bladder according to the anatomical ratio in a representative case.


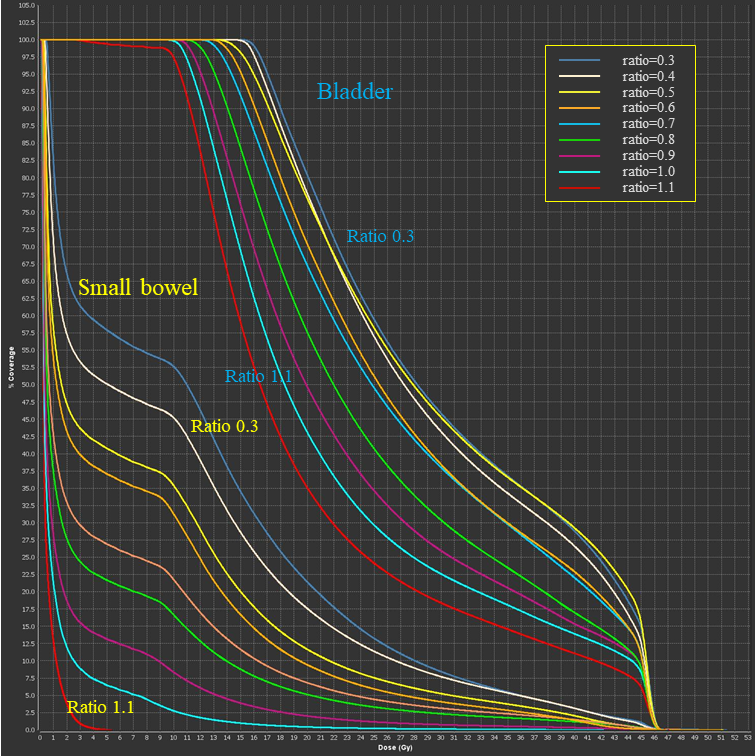

Supplement: Supplementary file 4 — Additional file 4: Figure S3. Dose-volume histogram of small bowel and bladder according to the anatomical ratio in a representative case. [file 12885_2020_7405_MOESM4_ESM.docx]
